# Anti-resorptive therapy in the osteometabolic patient affected by periodontitis. A joint position paper of the Italian Society of Orthopaedics and Traumatology (SIOT) and the Italian Society of Periodontology and Implantology (SIdP)

**DOI:** 10.1186/s10195-023-00713-7

**Published:** 2023-07-15

**Authors:** L. Landi, P. Tranquilli Leali, L. Barbato, A. M. Carrassi, N. Discepoli, P. C. M. Muti, G. Oteri, M. Rigoni, E. Romanini, C. Ruggiero, U. Tarantino, E. Varoni, N. M. Sforza, M. L. Brandi

**Affiliations:** 1 SIdP Panel, Via della Balduina 114, 00136 Rome, Italy; 2 SIOT Panel, Rome, Italy; 3 Private Practice Verona and Roma, Verona, Italy; 4 Department of Orthopedic Diseases, University of Sassari, Sassari, Italy; 5 Department of Experimental and Clinical Medicine, Research Unit in Periodontology and Periodontal Medicine, University of Florence, Florence, Italy; 6 Department of Biomedical, Surgical and Dental Sciences, University of Milan, Milan, Italy; 7 Department of Medical Biotechnologies, Unit of Periodontology, University of Siena, Siena, Italy; 8 Department of Biomedical and Dental Sciences and Morphofunctional Imaging, University of Messina, Messina, Italy; 9 RomaPro, Polo Sanitario San Feliciano, Rome, Italy; 10 Department of Medicine and Surgery, Gerontology and Geriatric Section, University of Perugia, Perugia, Italy; 11 Department of Clinical Sciences and Translational Medicine, “Tor Vergata” University of Rome, Rome, Italy; 12 Private Practice Bologna, Bologna, Italy; 13 Osservatorio Fratture da Fragilità, Via San Gallo 123, 50100 Florence, Italy; 14 Maxillo-Facial Surgery and Dental Unit, Fondazione IRCCS Ca’ Granda Ospedale Maggiore Policlinico, Milan, Italy

**Keywords:** Anti-resorptive therapy, Osteoporosis, Periodontitis, Periimplantitis, MRONJ

## Abstract

This joint report from the Italian Society of Orthopaedics and Traumatology (SIOT) and the Italian Society of Periodontology and Implantology (SIdP) aims for a consensus around the scientific rationale and clinical strategy for the management of osteoporotic patients affected by periodontitis who are undergoing anti-resorptive (AR) therapy to manage the risk of the occurrence of a medication-related osteonecrosis of the jaws (MRONJ). Osteoporosis and periodontitis are chronic diseases with a high prevalence in aging patients, and they share some of the same pathogenetic mechanisms based upon inflammation. Available evidence shows the relationship among osteoporosis, AR agents, periodontitis and implant therapy in relation to the incidence of MRONJ. Uncontrolled periodontitis may lead to tooth loss and to the need to replace teeth with dental implants. Tooth extraction and surgical dental procedures are recognized as the main risk factors for developing MRONJ in individuals taking AR therapy for osteometabolic conditions. Although the incidence of MRONJ in osteometabolic patients taking AR therapy may be as low as 0.9%, the increasing prevalence of osteoporosis and the high prevalence of periodontitis suggest that this potential complication should not be overlooked. Good clinical practice (GCP) guidelines are proposed that aim at a more integrated approach (prescriber, dentist, periodontist and dental hygienist) in the management of periodontitis patients undergoing AR therapy for osteometabolic disorders to reduce the risk of MRONJ. Dental professional and prescribers should educate patients regarding the potential risk associated with the long-term use of AR therapy and oral health behavior.

## Highlights

- AR drugs such as bisphosphonates (BPs), denosumab (DNB) and romosozumab (RMB) are very effective in the treatment of osteoporosis and in the prevention of fragility bone fractures.
- Periodontitis is a widespread infective inflammatory disease that is the major cause of tooth loss, and it is strongly connected with other systemic diseases, including osteoporosis.
- MRONJ is a serious and rare complication associated with the use of AR drugs in osteoporotic patients. The reported incidence is rather low and ranges between 0.01 and 0.9%, but may be higher in the presence of comorbidities.
- Periodontitis and MRONJ share some risk factors, such as diabetes, smoking, steroids, cardiovascular diseases and rheumatoid arthritis.
- The risk of developing MRONJ in a case of successfully treated periodontitis is much lower than the risk of fragility fracture in a high-risk person such as one with a previous fracture.
- Oral and periodontal conditions should be assessed before starting an AR therapy, and local intra-oral inflammation should be brought under control. Periodontal therapy is effective at reducing the risk of teeth extraction and therefore the need for major bone reconstructive intervention and implant placement.
- Control of periodontal inflammation should be achieved and maintained over time in osteoporosis-affected patients treated with AR.
- Peri-implant diseases rather than dental implant placement may be considered a trigger for MRONJ; for this reason, periodontitis and peri-implant inflammatory disease control and the inclusion of patients in a supportive periodontal program are critical.
- AR therapy should not be discontinued or deferred by the dentist unless done in accordance with the prescriber.
- The suspension of BP therapy is not recommended on a routine basis, as BP binds to the skeletal sites and continue to be released for months or years after treatment, with a long tail effect on bone metabolism.
- DNB administration should not be withdrawn because the rebound effect may increase the risk of bone fractures. A therapeutic window in which to perform dento-alveolar surgical procedures is suggested.
- It is advisable to calibrate the timing of dental extraction and surgical procedures between the dentist and prescribers according to the oral condition, the general health condition, and the time and type of AR drugs used.
- A more integrated approach between prescriber, dentist, periodontist and dental hygienist should be encouraged, particularly in the management of periodontitis-affected patients who are taking AR drugs for osteometabolic disorders.
- Prescribers and dentists must educate patients regarding the potential risk associated with long-term use of AR therapy.

## Introduction

Osteoporosis is a systemic skeletal disease characterized by altered bone metabolism and a subsequent higher fracture risk [55]. Anti-resorptive agents (AR) are effective in reducing the risk of fracture in osteoporotic patients [17]. Nevertheless, patients who have undergone AR treatment show an increased risk of developing an oral complication such as osteonecrosis of the jaw [10, 34, 35, 58].

Ever since Marx reported avascular necrosis of the jaw caused by injectable bisphosphonates (BPs) in 35 cancer patients and 1 osteoporotic patient, the term “bisphosphonate-related osteonecrosis of the jaw” (BRONJ) has been advocated for this condition [49]. In subsequent years, additional cases of osteonecrosis related to other drugs, including denosumab and anti-angiogenetic agents, were also reported [2, 57, 79, 82]. Accordingly, in 2014, for the first time, the American Association of Oral and Maxillofacial Surgeons (AAOMS) changed the definition of this complication to “medication-related osteonecrosis of the jaw” (MRONJ) to highlight that several drugs besides anti-resorptive agents and BPs can be responsible for its onset [66]. More recently, a new monoclonal antibody used for fracture prevention in osteoporotic women, i.e., romosozumab, was also associated with osteonecrosis of the jaw [67].

In 2022, the AAOMS updated the case definition of MRONJ [65] to include all the following elements:

1. Current or previous treatment with AR alone or in combination with immune modulators or antiangiogenic medications
2. Exposed bone or bone that can be probed through an intraoral or extraoral fistula(e) in the maxillofacial region and has persisted for more than 8 weeks
3. No history of radiation therapy to the jaws or metastatic disease in the jaws.

MRONJ may arise spontaneously, even though dento-alveolar surgery procedures are considered the main risk factor [30]. Moreover, the duration of AR therapy may also be related to an increased risk of MRONJ [46].

Periodontitis is a chronic multifactorial inflammatory disease characterized by the destruction of the supporting tissues of the tooth [18]. Untreated periodontitis may determine tooth loss [61]. Both periodontitis and osteoporosis share common pathogenetic mechanisms, and a bidirectional association can be hypothesized [47, 86].

Different manuscripts, position papers and guidelines [7, 13, 15, 65] have been proposed for the management of osteoporotic patients undergoing AR therapy who are in need of dento-alveolar surgery (i.e., tooth extraction; dental implants) and for the prevention and treatment of MRONJ; however, to the best of our knowledge, very few have specifically dealt with the clinical management of patients affected by both periodontitis and osteoporosis. This joint report of the Italian Society of Orthopaedics and Traumatology (SIOT) and the Italian Society of Periodontology and Implantology (SIdP) aims to clarify the possible implications of anti-resorptive (AR) therapy in osteoporotic patients at high risk of bone fragility fracture who are affected by periodontal diseases, including periodontitis and gingivitis. A focus on implant rehabilitation in this subgroup of patients will be also provided. The risk of developing MRONJ spontaneously or following oral and dental surgical procedures is assessed against the risk of skeletal bone fracture in patients on AR therapy. Then, clinical advice to prescribers, general dentists and periodontists is provided.

## Methodology

A panel of experts was identified by each society and on October 30th 2021, a first online meeting was held, and two co-chairs were chosen—one for each society (M.L.B. for SIOT, L.L. for SIdP). Panel members were asked to review the available evidence on the following topics: (1) osteoporosis and the use of AR agents, (2) MRONJ in patients treated with AR agents for osteoporosis, (3) periodontitis, (4) evidence of the association between periodontitis and osteoporosis, (5) periodontitis and MRONJ, and (6) dental implants and MRONJ. Each report was presented and discussed in five on-line meetings between February 1st 2022 and September 27th 2022, and a unanimous consensus was reached. Good clinical practice (GCP) guidelines were then elaborated by two members of the SIOT (E.R., M.L.B.) and three members of the SIdP (L.B., G.O., L.L.) and submitted to the panel in a two-round evaluation process. Algorithms to graphically describe the GCP guidelines considering the main clinical scenarios were also built. A plenary online session was held on November 9th 2022, and the document including the algorithms was approved unanimously.

## Current evidence and background

### Osteoporosis

Osteoporosis is a systemic skeletal disease characterized by a reduction in bone mass and quality (macro- and micro-architecture, material properties, geometry, and micro-damage), leading to bone fragility and a higher fracture risk [55]. The prevalence of osteoporosis is increasing worldwide with the aging of the global population [78]. The epidemiological impact of osteoporosis is impressive: in Italy, about 3.5 million women and 1 million men have osteoporosis, and, over the next 25 years the percentage of the total population that is over 65 will increase by 25% and hence a proportional increase in the prevalence of this condition is to be expected [55].

Osteoporosis is associated with a high risk of low-energy fractures of the hip, spine, proximal humerus and forearm. Hip fractures exert a more significant cost burden on healthcare services worldwide compared to other fracture sites [39]. Hip fracture commonly leads to long-term physical disability, reducing the quality of life and impairing the capacity to live independently and perform daily activities. The consequences of hip fractures also include depression, health comorbidities, and an increased risk of death [27]. In Italy, the number of older adults with hip fractures increased by 28.62% in 15 years [60].

Because bone loss occurs insidiously and qualitative skeletal changes are asymptomatic, osteoporosis is frequently diagnosed after the first clinical fragility fracture. At this point, anti-osteoporosis therapy is overdue but is required to prevent further fragility fractures in these high-risk patients. Therefore, it is pivotal to assess the osteoporosis and individual fracture risk early enough to prevent the first fracture, as well as to promptly identify and treat patients at very high risk of refracture because of a recent (within 12–24 months) osteoporotic fracture [85]. National and international guidelines have been implemented to address osteoporosis screening and secondary prevention in an evidence-based and cost-effective manner [12].

The pathogenesis of osteoporosis and fracture risk is multifactorial and depends upon several independent risk factors. Often, osteoporotic patients have risk factors leading to a bone mineral density (BMD) reduction and risk factors that are completely or partially independent of BMD, such as those affecting bone quality and extraskeletal factors. Overall, a low BMD, a medical history of fragility fracture, age, and a family history of osteoporosis are the main risk factors for osteoporotic fracture. However, several further factors and conditions may directly influence BMD, such as gender, calcium intake, physical activity, age of menopause, physical disability, environmental cues, alcohol consumption, drugs (e.g., steroids or diuretics), smoking, a low body weight, and vitamin D deficiency. In addition, comorbidity increases the individual fracture risk due to pathogenic mechanisms that affect both skeletal and extraskeletal pathways and can lead to falls and fractures. Ultimately, genetics have been shown to exert a strong influence on BMD and bone microarchitecture [78]. It has been stated that patients with multiple risk factors are at a higher risk of osteoporotic fracture than patients with a single risk factor, including an isolated reduction in BMD, due to their synergic effect, which exponentially increases the fracture risk.

Approved drugs for the treatment of postmenopausal osteoporosis include AR agents, such as BPs, denosumab (DNB), and selective estrogen receptor modulators (SERMs), the pure anabolic agent teriparatide, and the new bone‐forming and anti-resorptive agent romosozumab (RMB) (Table 1).

**Table 1:** The most common drugs available for the treatment of post-menopausal osteoporosis and other skeletal diseases

Category	Drug class	Drug name	Brand names
Anti-resorptive	N-bisphosphonates (N-BPs)	Alendronate Risedronate Zoledronate Ibandronate Pamidronate Minodronate	Binosto^®^, Fosamax^®^, Fosavance^®^ Actonel^®^, Actonel Combi^®^ Aclasta^®^ Zometa^®^ Bondronat^®^, Bonviva^®^, Iasibon^®^, Quodixor^®^ Aredia^®^
	BPs	Clodronate Etidronate	Bonefos^®^, Clasteon^®^, Loron^®^
	RANKL inhibitor monoclonal antibody	Denosumab	Prolia^®^, Xgeva^®^
Anabolic	PTH-related anabolic	Teriparatide	Forsteo^®^, Livogiva^®^, Movymia^®^, Osseffyl^®^, Sondelbay^®^, TeriparatideTeva^®^, Terrosa^®^
Bone builder	Anti-sclerostin monoclonal antibody	Romosozumab	Evenity^®^

## Pharmacologic therapy for osteoporosis

Management of osteoporosis requires a patient-centered and comprehensive approach to prevent bone fragility fractures. Thhis section focuses on the pharmacologic management of osteoporosis in relation to the development of MRONJ only.

- *Aminobisphosponates (BPs)*. BPs are analogs of inorganic pyrophosphate and inhibit bone resorption. They can block osteoclastic activity through a mechanism of action that depends on the presence or absence of an amino group in their chemical structure. All BPs developed so far for the treatment of skeletal diseases can reduce the bone turnover in a dose-dependent manner with a proportional increase in bone density and a decrease in fracture risk [17]. BPs can be classified into at least two groups with different molecular modes of action. The simpler non-nitrogen-containing BPs (such as etidronic acid and clodronate) can be metabolically incorporated into non-hydrolyzable analogues of ATP which interfere with ATP-dependent intracellular pathways. The more potent nitrogen-containing BPs (N-BPs), including pamidronate, alendronate, risedronate, ibandronate, minodronate, and zoledronate, are not metabolized in this way but inhibit key enzymes of the mevalonate/cholesterol biosynthetic pathway. N-BPs are used in a wide spectrum of indications such as the treatment and prevention of osteoporosis as well as the treatment of Paget's disease, multiple myeloma, and malignancy‐associated hypercalcemia [25]. For the purpose of this report, we will focus on N-BPs that are mainly approved for osteoporosis treatment and the prevention of osteoporotic fragility fractures.
- *Denosumab (DNB)* is a humanized monoclonal antibody capable of neutralizing RANKL, a cytokine that interacts with the RANK receptor on the membrane of pre-osteoclasts and mature osteoclasts, therefore affecting osteoclast recruitment, maturation, and survival [41]. Subcutaneous administration of DNB is followed by a reduction in osteoclastic bone resorption and, subsequently, a reduction in neoformative activity; for this reason, it is an anti-resorptive drug, like BPs. The most significant differences of DNB from BPs are (i) the length of pharmacological action, which ceases immediately upon the disappearance of the drug from circulation (usually within 5–6 months from subcutaneous administration), (ii) its uniform action on all skeletal structures irrespective of bone turnover, which results in greater pharmacological activity in the cortical bone, and (iii) the continuous increase in BMD associated with long-term therapy (up to 10 years), in contrast to what happens with other anti-resorptive drugs, which plateau in BMD after 3–4 years of therapy, particularly at the cortical level [55]. A noteworthy aspect of DNB treatment is that its discontinuation requires a rapid re-assessment of the patient’s fracture risk and the initiation of an alternative treatment by individuals at higher risk [42].
- *Romosozumab (RMB)* is a newly available humanized monoclonal antibody for osteoporosis treatment that binds and inhibits sclerostin, which, in turn, produces an early bone‐forming effect associated with a concomitant and sustained decrease in bone resorption [20]. The dual and opposite actions lead to increased bone mass, with higher BMD at the lumbar spine, total hip, and femoral neck after 12–36 months, and an improvement in microarchitecture parameters, with both contributing to a significant reduction in vertebral, nonvertebral and clinical fractures in postmenopausal women with severe osteoporosis [72].
- *Teriparatide*, a pure anabolic drug, is the active fragment (recombinant human PTH 1–34) of parathyroid hormone that markedly stimulates bone formation. The effect of teriparatide on trabecular BMD is significantly greater than that obtained with bisphosphonates, and significantly reduces the frequency of vertebral and non-vertebral fracture in post-menopausal women [90].

## MRONJ in patients treated with AR agents for osteoporosis

At present, the pathophysiology of this condition has not been fully determined, and there is much debate about the mechanisms by which AR agents may induce necrosis in the jawbone [70]. Recently, the updated AAOMS position paper on MRONJ [65] reported on the pathophysiology of this condition, which remains multifactorial in nature. Several hypotheses have been proposed, such as bone remodeling and angiogenesis inhibition, innate or acquired immune dysfunction, genetic predisposition, as well as inflammation or infection [4], with the latter seeming to play a major role when combined with anti-resorptive agents in inducing MRONJ [70]. The incidence of MRONJ in osteoporotic patients treated with AR has been reported to be much lower compared to oncologic patients. Indeed, according to the AAOMS, the risk of MRONJ among osteoporotic patients exposed to BPs, DNB, and RMB is low [65]; the incidence ranges from 0.02% to 0.05%, nearly 10 times less than what has been reported for cancer patients, for whom the incidence may be as high as 8% [88], which is particularly related to the co-administration of AR with glucocorticoids or immunosuppressive agents [9].

However, recent large retrospective studies reported higher figures for MRONJ, with a range from 0.55% [29] to as high as 0.9% [28]. Indeed, the incidence seems to rise in cases of autoimmune disorder (7.2%) and rheumatoid arthritis (13%) [28]. Patients exposed to DNB for osteoporosis seem to carry a higher risk of MRONJ compared to BPs, with an incidence that ranges from 0.04% to 0.68% [29, 65, 83],). In the case of RMB, rare cases of MRONJ have been reported in RCTs [67], and the overall incidence remains similar to BPs (0.03–0.05%) [65]. For the anabolic drug teriparatide, MRONJ has not been reported as a complication. Conversely, the drug was used for its anabolic action in isolated cases in an attempt to cure the oral complications linked to anti-resorptive therapy, enhancing bone remodeling and accelerating osseous wound healing [8, 43, 52]

MRONj can be considered a rare albeit serious complication although the population who may be exposed to the risk of developing it is progressively increasing. The number of patients being treated with AR for osteometabolic disorders is increasing, and the total number of patients outweighs the number of cancer patients. In Italy, about 4.5 million people are diagnosed with osteoporosis, 80% of whom are post-menopausal women. In 2021 there was a 20.9% increase in the consumption of DNB, while the prescription of BPs reduced (− 7.6%) [92]. Despite the low incidence of MRONJ, we cannot ignore the fact that a higher number of patients may be exposed to the risk of developing this complication, so a thorough oral examination is recommended—with particular emphasis placed on periodontal screening—before starting AR therapy and periodically after its initiation [66].

## Periodontitis

Periodontitis is a chronic inflammatory disease characterized by the progressive destruction of the supporting tissues of the tooth: gum, alveolar bone, root cement and periodontal ligament. Periodontitis is a multifactorial disease characterized by the presence of bacterial plaque (dental biofilm) as the causative factor. Genetic predisposition, obesity and a pro-inflammatory diet are other risk factors/indicators that contribute to disease development and progression [18]. Furthermore, periodontal disease has been strongly associated with diabetes in a bidirectional manner [24, 68].

Periodontitis is the sixth most frequent chronic non-communicable disease, affecting nearly 800 million people worldwide [21]. In Italy, one in five adults is affected by the most severe form of periodontal disease, which can lead to tooth loss [5, 61] with a negative impact on chewing, aesthetics and quality of life [63]. Periodontitis typically arises between the ages of 30 and 40, although even young individuals can be affected, albeit rarely [51]. Recently, a new classification of periodontal diseases has been published. Periodontitis includes four different stages of increasing severity, stage IV being the most severe form. There are three grades of risk of disease progression: A, B and C, where grade C carries a higher risk of progression compared to grades A and B [80]. Males, smokers, and those with poor oral hygiene and a family history of periodontitis are more frequently affected [26]. Host–parasite interactions in periodontitis patients promote proinflammatory molecule local production, which can, through epithelial gingival lesions of the periodontal pocket, reach the bloodstream and contribute to the increase in the global inflammatory load of the patient [36].

Periodontitis, owing its systemic inflammatory impact, belongs to the group of non-communicable diseases (NCDs), and it has been associated with other systemic diseases, such as cardiovascular diseases, diabetes, rheumatoid arthritis and other conditions [23]. Low-grade chronic inflammation and shared common risk factors are the pathogenic background that sustains such a link. Moreover, evidence suggests that related periodontal treatment can actively decrease the pro-inflammatory mediators of the systemic burden [18, 23, 53, 68]. Recently, osteoporosis and periodontitis have been related bidirectionally: patients with osteoporosis may be at a higher risk of developing periodontitis, and individuals affected by periodontal diseases may have a higher likelihood of developing osteoporosis [86].

If the periodontal inflammation remains untreated, it could progress to the destruction of the alveolo-dental ligament, leading to partial or complete loss of the dentition, with possible masticatory and aesthetic impairments.

With such a clinical scenario, early diagnosis and treatment of gingival inflammation (gingivitis) represents a keystone for primary and secondary prevention of periodontitis. One condition that precedes periodontitis development is gingivitis, which can affect nearly 60% of the adult population [45]. This condition is often underestimated or even mistakenly considered a normal condition. Therefore, gingival bleeding is not recognized as an early sign of inflammation, and often the patient does not refer promptly to the dentist or to the dental hygienist [19].

Unlike dental caries, periodontitis causes not only the loss of a tooth, but also the loss of the surrounding supportive tissues, including severe alveolar bone loss and gingival recessions. The resulting potentially extensive anatomical defects can ultimately make the oral rehabilitation therapy, especially when including dental implants, significantly more complex.

Periodontal therapy includes a stepwise approach with four steps. Steps 1 and 2 focus on the control of inflammation and systemic risk factors, such uncontrolled diabetes and smoking. Step 3 includes non-surgical re-treatment of residual pockets, followed in specific cases by surgical treatment. Step 4 provides a periodontal supportive care program (PSC) that is critical to maintaining periodontal health over time. After periodontal therapy, the clinician can face two different levels of disease: stable periodontal disease with a reduced periodontium (BOP < 10% and PPD < 5 mm) or periodontal disease remission/control. The latter is defined as a period in the course of the disease when symptoms become less severe but may not be fully resolved. From step 1 onward, it’s mandatory for the patient to achieve proper biofilm control at home, which commonly translates into an Oral Hygine Index (OHI-I)  of < 20% [59, 69].

## Focus questions

### What is the evidence for the association between osteoporosis and periodontitis?

Both periodontitis and osteoporosis are diseases that show alterations of the bone metabolism which are closely associated with inflammation and aging [89]. The multifactorial nature of the host response to periodontal disease led to the hypothesis that periodontitis could be a risk factor for the progression of osteoporosis and vice versa [47, 86].

Suggested mechanisms underlying the former link are the disruption of the homeostasis concerning bone remodeling, the hormonal balance, and the resolution of the inflammatory pathway [91].

Patients with periodontitis exhibit high levels of inflammatory mediator that contribute to the global inflammatory burden, whose systemic effects may sustain the inflammatory cascade related to the osteometabolic event [23, 53].

On the other hand, the reduced bone mineral density caused by osteoporosis can jeopardize the alveolar bone resorption already settled as a result of the inflammatory process triggered by the dysbiosis between the dental biofilm and the host’s immune system [86]. The high prevalence of periodontitis and osteoporosis in the aging population may therefore jeopardize the successful control of both diseases.

Chronic inflammation contributes to the risk of osteoporosis. From this perspective, the clinical resolution of periodontal inflammation can play a role in reducing systemic inflammatory processes, and periodontal treatment protocols may act as a tool in the control of systemic health [23]. Treatments aiming at lowering the systemic inflammation may improve bone mineral density and reduce the risk of fragility-related fractures [89].

Periodontal therapy has recently been updated and a stepwise approach has been suggested. This approach has proven to be very effective in maintaining oral health and reducing the risk of tooth loss along with all of its related potential sequelae, including the need for major bone reconstructive procedures and dental implant surgery [38, 69].

Therefore, it seems reasonable to suggest that all individuals with bone fragility, those who are post-menopausal, and those who carry other comorbidities should undergo periodontal evaluation regardless of the need to initiate AR therapy for osteometabolic disorders.

Periodontal stability should be achieved and possibly maintained throughout our lives. As the risk of MRONJ increases over time, the strategic role of periodontal supportive care (step IV) [69] is evident and strongly suggested.

### Is periodontitis a risk factor for MRONJ?

The biological rationale behind this relationship has been studied in animal models [3, 74]. The combined effect of local soft and hard tissue traumatism (i.e., tooth extraction) and the resulting inflammatory alteration/impairment might represent the current MRONJ etiopathogenic model [32, 44]. The understanding of such a complex clinical phenotype is based on the impairment of bone and connective tissue homeostasis fostered by AR intake. Indeed, AR agents decrease the osteoclastic activity, therefore limiting bone resorption and remodeling. This phenomenon prolongs bone exposure to a local environment with periodontitis, which is characterized by oxidative stress, endotoxemia and proinflammatory mediators, thus precipitating cellular and molecular toxicity and ultimately the onset of local necrosis [40]. As regards connective tissue homeostasis, a recent piece of evidence obtained in vitro demonstrated that BP intake can jeopardize the mitotic activity of human gingival fibroblasts [77].

Several epidemiological studies have identified periodontal disease as a risk factor for MRONJ. A recent systematic review has explored the link between periodontal disease and MRONJ in case–control and retrospective cohort studies [48]. Despite the great heterogeneity of case definitions regarding periodontal disease—based on both clinical and radiographic records—the risk of PD in MRONJ-affected patients versus controls was significantly greater (RR 2.75, 95% CI 1.67–4.52). A pooled analysis of both probing pocket depth (PPD) and clinical attachment level (CAL) showed a non-statistically-significant difference between the MRONJ patients and the controls.

Periodontitis could also be considered a functionally linked risk factor that predisposes to MRONJ development after tooth extraction in the presence of AR therapy, including BPs or DNB. The periodontal condition was shown to have a significant impact on the risk of developing MRONJ after adjusting for BP use [16]. Osteonecrosis may be associated with the duration of the preexisting pathological periodontal inflammatory conditions. An animal experiment showed that a periodontal cleaning—in line with periodontal maintenance therapy in humans, which targets the debriding of accretions from sites afflicted by periodontitis—improves periodontal conditions and reduces MRONJ prevalence in zoledronate-treated rats [16].

Although MRONJ is an uncommon complication in subjects taking BP or DNB for the treatment of osteoporosis, its effects on the oral tissues can be severe. It should be mentioned that much of the currently available information on this adverse effect has been obtained mainly from observational studies and not from clinical trials. Therefore, it is not surprising that the position of the scientific community on certain critical points regarding the prevention of MRONJ is sometimes controversial, such as on the use of antibiotic prophylaxis in the management of surgical intraoral procedures [11], the opportunity to temporarily suspend drug administration [62] or the selection of a therapeutic window to perform dento-alveolar surgical procedures in the case of DNB therapy [14].

### Are dental implants a risk factor for MRONJ?

Evidence for the effects of implant placement in osteoporotic patients under AR therapy is still sparse, and proper designs (randomized clinical trials, cohort studies) are lacking.

Most of the reported cases of peri-implant MRONJ were not associated with implant placement (dentoalveolar surgical procedure), but evolved de novo on previously osseointegrated implants [31, 71].

It seems that low-dose oral BP intake for osteoporosis treatment does not, in general, compromise implant therapy. Moreover, AR therapy seems to affect neither the survival rate of dental implants nor implant-related complications/failures in comparison to implants placed in patients without a BP intake [75].

However, some studies (mainly case reports) support the association of peri-implant biological complications, such as peri-implantitis, with MRONJ occurrence, particularly in periodontitis patients, patients who have been undergoing AR therapy for more than 5 years, and those who carry comorbidities [71, 75].

Considering the need for implant placement in patients under AR therapy, robust data are lacking and the literature is controversial, so caution is needed in case there is a longer duration of therapy or steroid use [33, 65]. In a systematic review, Granate-Marques and coworkers reported an elevated MRONJ risk associated with implants placed in the posterior jaw if the duration of bisphosphonate therapy exceeded 3 years and if the patients were receiving systemic corticosteroids [33]. More recently, two further systematic reviews concluded that patients with a history of BP treatment are at risk of developing MRONJ following implant placement (“implant-surgery-triggered” MRONJ), while those with a history of denosumab for osteoporosis show a negligible risk [56, 73]. However, data were based on case series and low-quality observational studies, making risk estimates very difficult. In the case of DNB, in particular, a single case series with 212 osteoporotic patients was considered: DNB was prescribed (60 mg administered every 6 months), and there was one reported case of MRONJ [83]. Considering all these limitations, the AAOMS suggest that DNB has a similar level of risk to BPs [65].

Overall, peri-implantitis rather than the surgical procedure for implant placement itself might constitute a main trigger factor for peri-implant MRONJ. However, dental clinicians should consider that there is a general lack of high-quality evidence regarding the safety of placing implants in patients with a history of anti-resorptive or anti-angiogenic medications, and they should use caution when planning dental implants in patients under AR therapy [56, 73]. Obtaining an adequate patient-specific informed consent which includes the low risk of MRONJ as well as early and late implant failure is always recommended before implant placement. Nonetheless, these patients should be placed on a regular long-term recall maintenance program. Table 2 summarizes the main risk factors for MRONJ development at both local and systemic levels in order to identify a patient-related risk profile.

**Table 2:** Identification of the patients’ risk profile for MRONJ

Systemic risk factors	Local risk factors	
	Periodontally related factors	Non-periodontally related factors
AR therapy > 3 years	Poor oral hygiene (PlI > 30%)	Root/cervical caries
Age > 65	Generalized gingivitis (BOP > 30%)	Periapical endodontic lesion
Previous history of chemotherapy	Untreated/uncontrolled periodontitis (PD > 4 mm, BOP > 20%, progressive bone loss)	Partially impacted teeth
Diabetes	Uncontrolled periodontitis patient treated with dental implants	Fractured teeth/residual roots
Steroid therapy	Presence of peri-implantitis (PD > 6 mm, BOP/PUS, RX bone loss > 3 mm)	Traumatic ulcerations in the presence of a removable partial denture
Kidney diseases		
Cardiovascular diseases		
Smoking		
Rheumatoid arthritis		

## Conclusions

The SIOT-SIdP panel discussed and elaborated expert opinions and recommendations based on the quality level of the existing evidence and focusing only on the specific cohort of patients affected by periodontal diseases while taking AR therapy for osteometabolic disorders. For oncologic patients in AR therapy, and for all other dental and oral conditions, the panel suggests referring to the SIOCMF-SIPMO guidelines [13, 15].

### Good practices in periodontitis patients under AR therapy for osteoporosis

Primary prevention of MRONJ could be significantly enhanced by increased collaboration between prescribers and dentists. The researchers that have evaluated this degree of cooperation have highlighted a low level of connection between these two professions [87]. SIOT and SIdP believe that to achieve better control of this uncommon but potentially severe pathology, it is essential to promote greater and more effective partnership between prescribers and dentists. Dentists should not suggest to the patient that they should stop taking the AR therapy, and tight collaboration between the dentist and the prescriber should be ensured. AR therapy suspension or a deferral of DNB administration should be decided by the prescriber only.

### Recommendations for the prescribers (expert opinion statements based on existing evidence)

1. **Patients about to start AR therapy:** Prescribers should educate patients about the long-term effect of AR therapy and about the risk of MRONJ. Patients should therefore be referred for a complete dental and periodontal examination before starting the treatment. Prescribers may ask the patient to use the SIdP app* GengiveInForma* (https://www.sidp.it/app/gengiveinforma/) to evaluate the risk that they have periodontal diseases. Patients with periodontal disease who have comorbidities (diabetes, CVD, steroids, rheumatoid arthritis, obesity, etc.) or a history of chemotherapy or who are current smokers should be considered at higher risk of developing MRONJ in the case of oral surgical procedures. Periodontal therapy should be carried out before AR therapy is initiated. LOW RISK OF MRONJ DEVELOPMENT.
2. **Patients who have been on AR therapy for less than 3 years**: Patients should be encouraged to visit the dentist regularly for a periodical check-up and for oral and periodontal health maintenance. Any major oral surgical procedure (i.e,. periodontal surgery, dental extraction, dental implants, etc.) should be postponed until periodontal inflammation is under control, there is minimal bleeding on probing, and proper biofilm control is achieved by the patient. LOW RISK OF MRONJ DEVELOPMENT.
3. **Patients who have been on AR therapy for more than 3 years:** A higher risk for MRONJ may be expected for these patients. Patients with periodontitis should attend a personalized supportive periodontal care program to minimize the risk of periodontal disease recurrence, consequently reducing the risk of increasing the number of hopeless tooth extractions and developing peri-implantitis. It seems wise, given the lack of evidence, to also include in this category patients who have been in AR therapy for > 3 years and have discontinued the treatment, in particular those who were using BP medications. Furthermore, if dental extraction for non-periodontal reasons is indicated and undeferrable, an increased risk of developing a complication may be considered, and the patient should be managed accordingly. INCREASED RISK OF MRONJ DEVELOPMENT.

### Recommendations for the dentist and the periodontist (expert opinion statements based on existing evidence)

Dentists are encouraged to carry out a complete oral examination, with a particular emphasis on periodontal conditions, following the EFP-SIdP guidelines [69], https://snlg.iss.it/wp-content/uploads/2021/10/LG-369-SIdP-parodontite.pdf). A recent orthopantomography may be indicated for a complete evaluation. Periodontal diagnosis is simple and effective and is based upon the recording of the periodontal probing depth (PD), the bleeding on probing index (BOP) and the radiographic bone loss (RBL). The oral hygiene index (the level of home biofilm control by the patient) must also be assessed. Staging and grading must then be defined, so that periodontal therapy can be established accordingly, and control of inflammation obtained. Whenever possible, it is suggested that any surgical procedures required prior to initiating AR therapy should be performed [13, 15, 65, 70]. Maintaining high standards of oral hygiene and dental care is pivotal to preventing dental diseases that may require extractions or other dentoalveolar surgeries. Step 4, i.e., supportive periodontal care, should be assured for all these patients. Periodontitis-affected patients with one or more comorbidities (i.e., diabetes, cardiovascular diseases, etc.) must be considered at increased risk of MRONJ compared to other osteoporotic patients and should be treated accordingly. In smokers, smoking cessation should always be advocated. In these patients, periodontal health should be ensured, and systemic disease controlled before any elective oral surgical procedure is considered. Although the risk of MRONJ cannot be completely eliminated, these preventive procedures are recommended [13, 15, 65, 70]. If a dentoalveolar surgical procedure is unavoidable, patients should be informed of the associated risks.

**Fig. 1:**
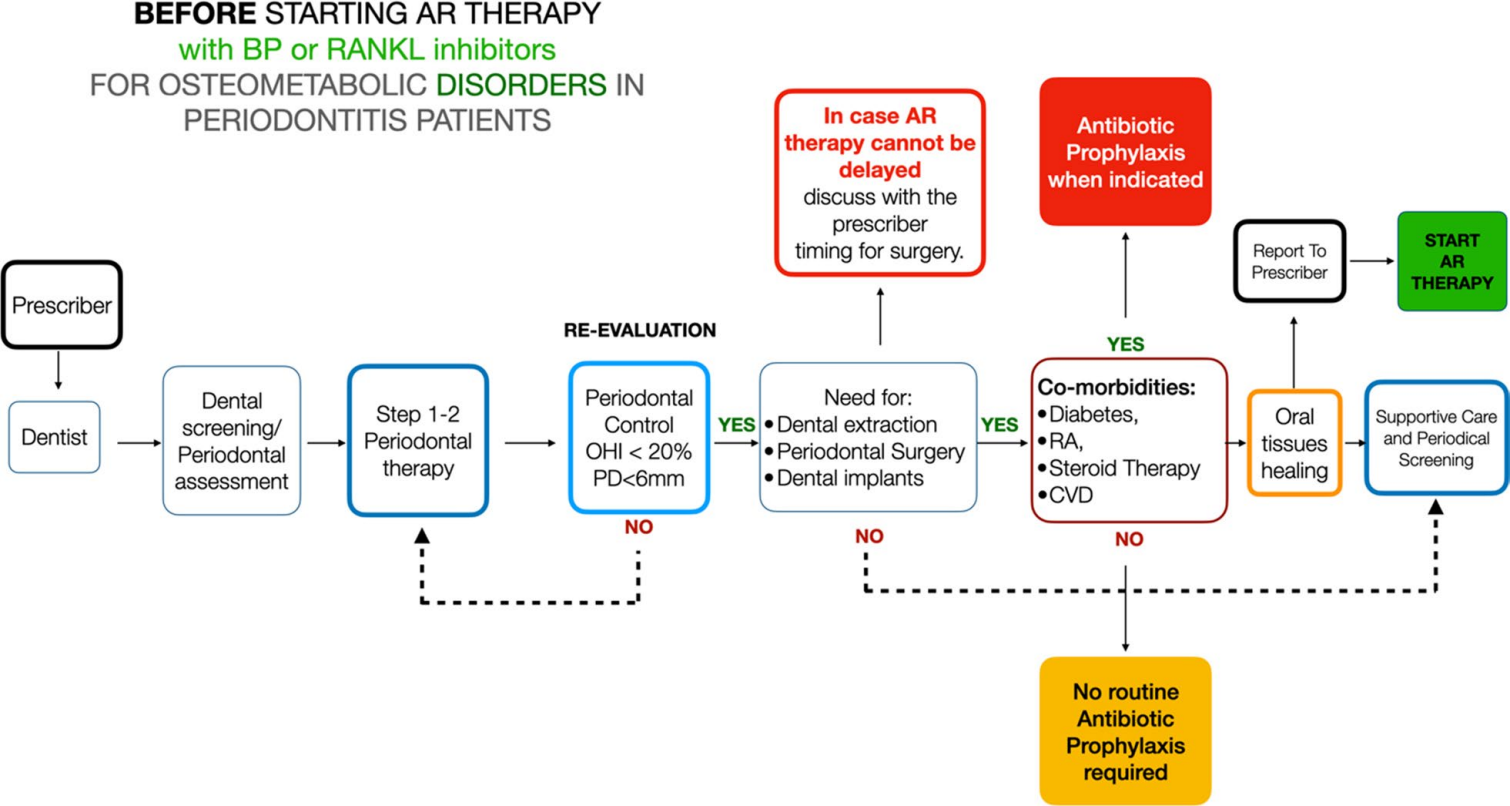
Proposed algorithms for patients who are about to start AR therapy for osteometabolic disorders

**Fig. 2:**
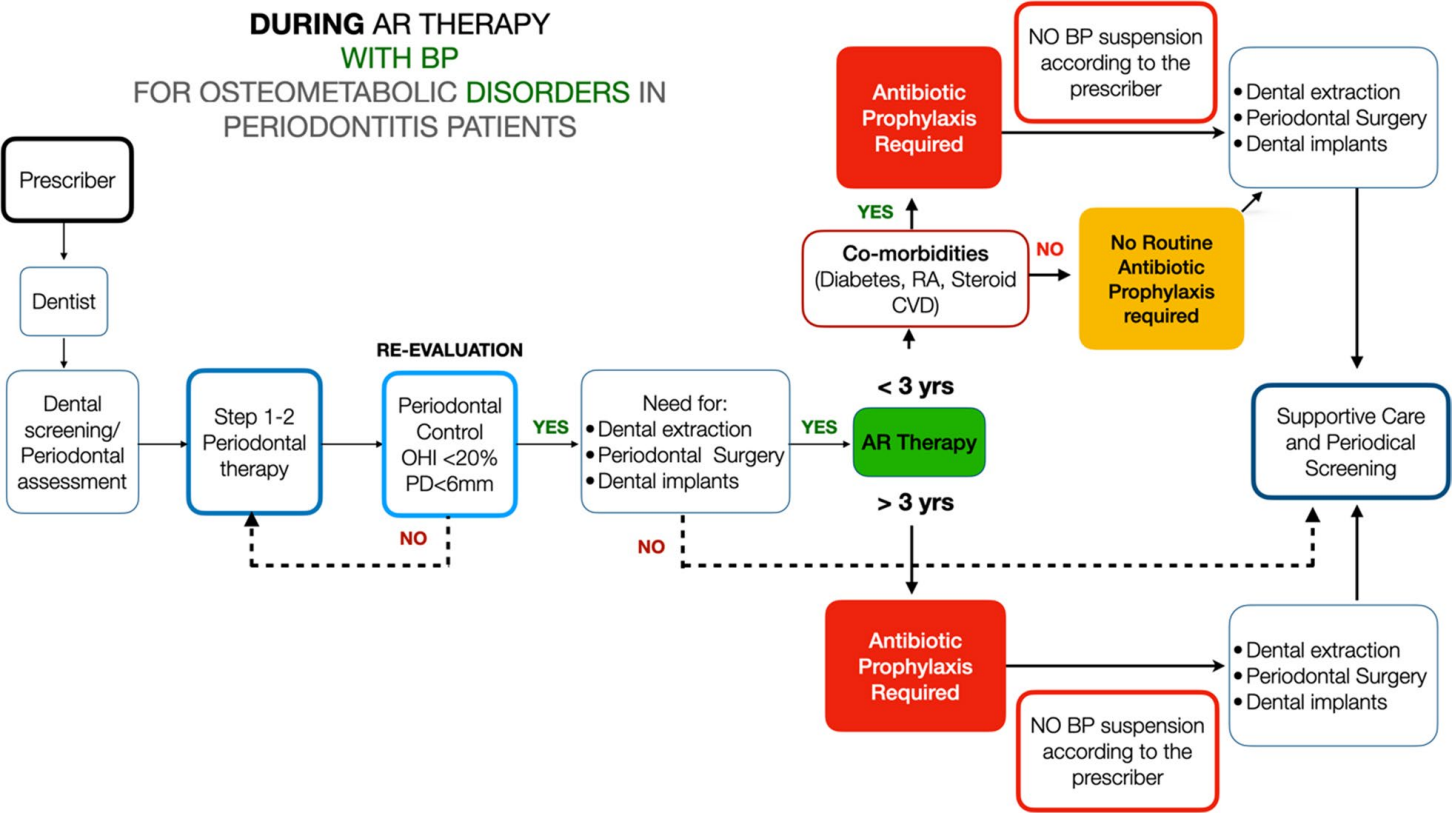
Proposed algorithms for patients who are in treatment with bisphosphonates

**Fig. 3:**
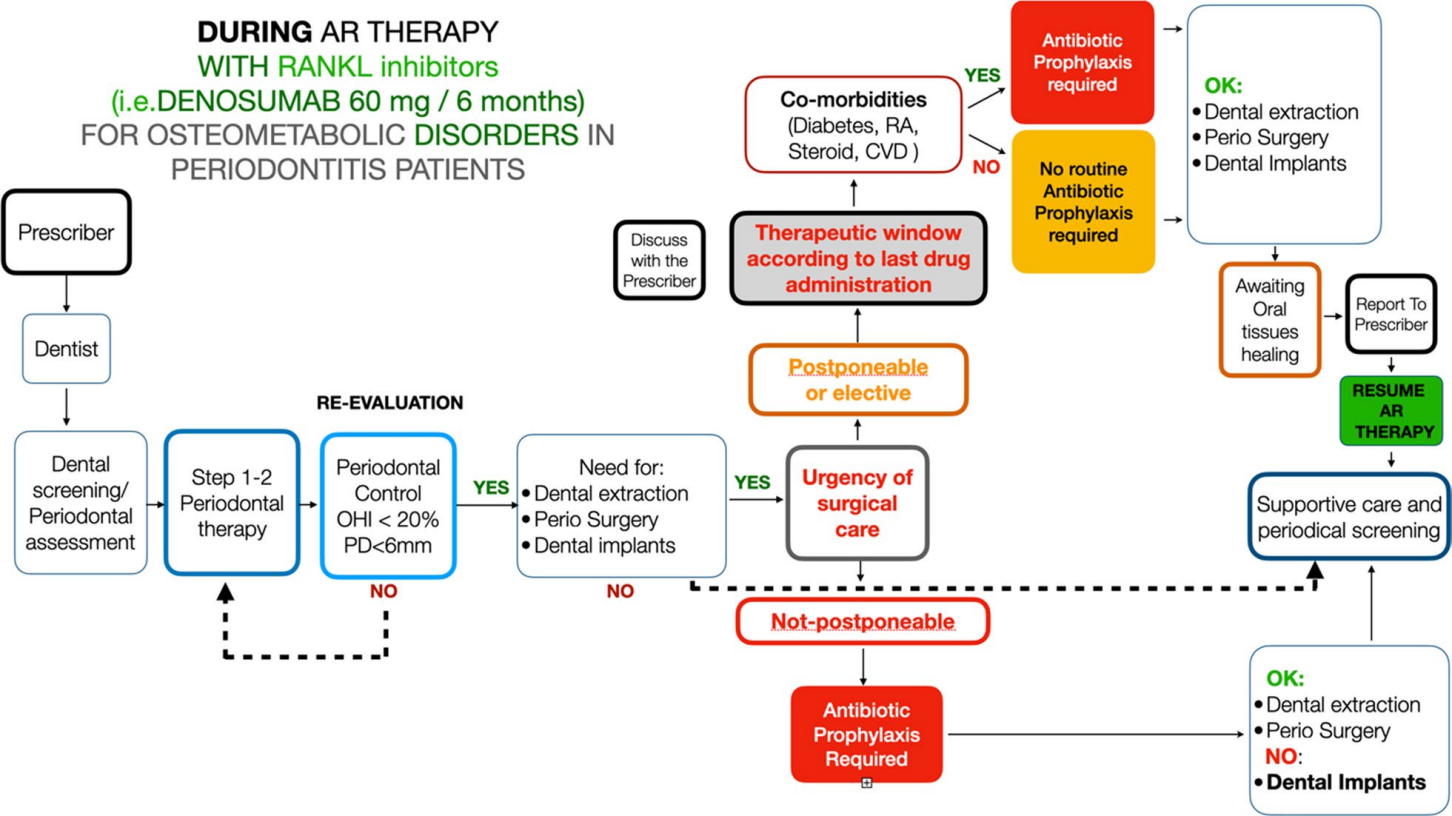
Proposed algorithms for patients who are in treatment with denosumab

Three main clinical scenarios may be encountered by the dentist:

1. **Patient waiting to start an AR therapy for osteometabolic disorders** (Fig. 1): It is suggested that patients should be referred by the prescriber to the dentist to get a thorough dental examination. If periodontal disease is diagnosed, a stepwise treatment according to the level of severity and complexity of periodontitis (i.e., the stage and the grade) should be implemented, following the clinical guidelines for periodontal treatment. The patient should be placed in a supportive periodontal care program (SPC) to prevent future periodontal disease progression. The same applies to peri-implant diseases, i.e., mucositis and peri-implantitis. Surgical procedures or dental extraction should be carried out preferably before AR therapy is established, and they may be carried out following standard procedures. No routine systemic antibiotic prophylaxis is required unless other comorbidities are present. LOW RISK OF MRONJ DEVELOPMENT
2. **Patients taking BP for osteometabolic disorders** (Fig. 2): If the patient was not screened before for periodontitis, they should be referred to the dentist/periodontist for a complete evaluation. If periodontitis is detected, steps 1 and 2 of periodontal therapy must be carried out until periodontal stability is successfully obtained. No antibiotic prophylaxis is required at this stage. If further treatment is indicated (step 3—surgical periodontal therapy), it can be subsequently carried out. Hopeless teeth may be extracted and/or osseointegrated dental implants may be placed, following a standard surgical protocol, provided periodontal control is successfully achieved. Patients taking AR therapy for > 3 years seem to have an increased risk of developing MRONJ compared to those with < 3 years of AR therapy. For this reason, even in the absence of clear evidence, it seems wise to suggest antibiotic prophylaxis to carry out dento-alveolar surgeries in this category of patients. For those who have taken AR therapy for < 3 years, antibiotic prophylaxis may not be used on a routine basis but according to a specific clinical scenario. In the presence of comorbidities, antibiotic prophylaxis is required regardless of the duration of AR therapy. Although clear evidence is missing, the same approach may be suggested for those patients who have been in AR therapy with BP for > 3 years and have discontinued the treatment. If antibiotic prophylaxis is indicated, amoxicillin 1 g three times a day alone or with clavulanic acid has usually been suggested. Also, the combination of amoxicillin 1 g three times a day and metronidazole 500 mg t/d has been suggested. There is no agreement regarding the postoperative administration period, which could be between 5 and 17 days. This decision should be taken by the dentist in relation to the level of risk to the patient and to the patient’s healing process after surgery [13, 15]. A drug holiday has not proven to be effective in the prevention of MRONJ in the case of BP therapy [37, 54, 65], and therefore cannot be recommended on a routine basis. BPs bind to the skeletal sites and are accumulated over time, leading to a reservoir that continues to be released for months or years after treatment is stopped, with a long tail effect on the prevention of fracture, particularly for alendronate and zoledronate [1, 84]. For example, the amount of alendronate released from bone over the next several months or years after a 10-year treatment period with alendronate would be equivalent to taking one-quarter of the usual dose [64]. This makes BP suspension virtually ineffective at reducing the risk of developing MRONJ. BP therapy < 3 years : LOW RISK of MRONJ DEVELOPMENT. BP therapy > 3 years: INCREASED RISK of MRONJ DEVELOPMENT.
3. **Patients taking AR therapy with RANKL inhibitors** (Fig. 3): Although a low risk of MRONJ may be anticipated for these patients, it is mandatory to obtain periodontal health to reduce the need for dental extraction and/or major surgical procedures. A periodontal evaluation should be carried out, and steps 1 and 2 of periodontal therapy must be implemented. Teeth extractions or oral surgery procedures, including dental implants, should be delayed until periodontal control has been established. In all cases where dental extractions or dento-alveolar surgical procedures cannot be postponed, antibiotic prophylaxis is required. DNB administration should not be withdrawn because of the well-known rebound effect, with an increased risk of fragility fractures after suspension [22, 81]. A single subcutaneous dose of 60 mg reaches its peak serum concentration within 4 weeks and declines over a period of 4–5 months to a level below assay limits. Repeated dosing every 6 months did not result in drug accumulation, and the DNB level at 24 months was comparable to baseline [6, 50, 76]. Therefore, in cases where surgical procedures or dental extractions may be delayed, a therapeutic window approach is suggested. In such cases, it seems highly recommendable to perform oral surgical procedures 3–4 months after the last DNB administration, while a new dose may be administered 6–8 weeks after tooth extraction or the surgical procedure and according to the healing process of the oral wound [65]. A safer option could be to delay dento-alveolar surgery until the 5th month after the last DNB dose [14]. It is part of good clinical practice to communicate with the bone specialist (drug prescriber) and possibly discuss the feasibility of further delaying the following denosumab dose by 1 month [14]. In this scenario, antibiotic prophylaxis is required only in the presence of comorbidities. LOW RISK OF MRONJ DEVELOPMENT.

## Data Availability

Not applicable.
